# Particle size dependent deposition and pulmonary inflammation after short-term inhalation of silver nanoparticles

**DOI:** 10.1186/s12989-014-0049-1

**Published:** 2014-09-17

**Authors:** Hedwig M Braakhuis, Ilse Gosens, Petra Krystek, John AF Boere, Flemming R Cassee, Paul HB Fokkens, Jan Andries Post, Henk van Loveren, Margriet VDZ Park

**Affiliations:** Department of Toxicogenomics, Maastricht University, PO Box 616, Maastricht, 6200 MD the Netherlands; National Institute for Public Health and the Environment (RIVM), PO Box 1, Bilthoven, 3720 BA the Netherlands; Philips Innovation Services, High Tech Campus 11, Eindhoven, 5656 AE the Netherlands; Institute of Risk Assessment Sciences, Utrecht University, PO Box 80.163, Utrecht, 3508 TD the Netherlands; Cellbiology, Biology Department, Faculty of Science, University of Utrecht, PO box 80.056, Utrecht, 3508 TB the Netherlands

**Keywords:** Nanoparticles, Inhalation exposure, Pulmonary toxicity, Cellular uptake, Dissolution

## Abstract

**Background:**

Although silver nanoparticles are currently used in more than 400 consumer products, it is not clear to what extent they induce adverse effects after inhalation during production and use. In this study, we determined the lung burden, tissue distribution, and the induction and recovery of adverse effects after short-term inhalation exposure to 15 nm and 410 nm silver nanoparticles.

**Methods:**

Rats were nose-only exposed to clean air, 15 nm silver nanoparticles (179 μg/m^3^) or 410 nm silver particles (167 μg/m^3^) 6 hours per day, for four consecutive days. Tissue distribution and the induction of pulmonary toxicity were determined at 24 hours and 7 days after exposure and compared with the internal alveolar dose. Presence of silver nanoparticles in lung cells was visualized by transmission electron microscopy (TEM).

**Results:**

Exposure to 15 nm silver nanoparticles induced moderate pulmonary toxicity compared to the controls, indicated by a 175-fold increased influx of neutrophils in the lungs, a doubling of cellular damage markers in the lungs, a 5-fold increase in pro-inflammatory cytokines, and a 1.5-fold increase in total glutathione at 24 hours after exposure. All the observed effects disappeared at 7 days after exposure. No effects were observed after exposure to 410 nm silver particles. The internal alveolar mass dose of the 15 nm nanoparticles was 3.5 times higher compared to the 410 nm particles, which equals to a 66,000 times higher particle number. TEM analysis revealed 15 nm nanoparticles in vesicles and nuclei of lung cells, which were decreased in size to <5 nm at 24 hours after exposure. This demonstrates substantial dissolution of the silver nanoparticles.

**Conclusion:**

The results show a clear size-dependent effect after inhalation of similar mass concentrations of 15 nm and 410 nm silver (nano)particles. This can be partially explained by the difference in the internal alveolar dose between the 15 nm and 410 nm silver (nano)particles as well as by a difference in the release rate of silver ions.

**Electronic supplementary material:**

The online version of this article (doi:10.1186/s12989-014-0049-1) contains supplementary material, which is available to authorized users.

## Background

Nanomaterials are used in a rapidly increasing number of products including consumer products [1]. However, there are concerns these nanomaterials might introduce health risks upon occupational and consumer exposure. Although many nanomaterials are produced, handled and present in fluids, aerosolization may occur during energetic processes, such as vortexing, weighing, sonication, mixing and blending [2]. In addition, nanomaterials may be released from their matrix during its use. Therefore, inhalation is considered a relevant route of exposure [2,3]. Currently, silver nanoparticles are the most common nanoparticles mentioned in product descriptions. According to the Nanotechnology Consumer Products Inventory, silver nanoparticles are currently claimed to be used in more than 400 consumer products [1]. Silver nanoparticles have antimicrobial activity and are used in food packaging material, food supplements, odour-preventing textiles, cosmetics, kitchen utensils, toys, electronics, wound dressings, and room sprays [1,4]. Silver (nano)particles and released ions exert antimicrobial properties by binding to sulphur- and phosphorous-containing biomolecules such as proteins and DNA, thereby potentially also causing damage to mammalian cells [5-9]. Besides worker exposure, the extensive use of silver nanoparticles in products might also lead to consumer exposure by inhalation of the silver nanoparticles that are used in spray applications. Although silver nanoparticles are currently claimed to be used in many products, it is not clear to what extend they induce adverse effects after inhalation during production and use.

Silver nanoparticles have been studied in several *in vivo* inhalation studies showing diverse outcomes. Some studies showed no induction of adverse effects [10,11], while other studies reported adverse effects varying from a minimal inflammatory response to the presence of inflammatory lesions in the lungs [12-15]. Regarding the tissue distribution of the silver nanoparticles, some studies report a dose-dependent increase in the silver concentration in the lungs and in the liver [11,13,15]. Two of these studies report a rather high amount of silver detected in the brain and the olfactory bulb [11,15], causing concerns that silver nanoparticles might induce toxicity in the brain.

The before mentioned inhalation studies show that silver nanoparticles can induce pulmonary inflammation and can decrease lung function, depending on the exposure time and dosage [10-15]. However, all of these studies focused on a single particle size. The previous studies did not take particle size and surface area into account as explaining variable, whereas these affect the internal dose and the interaction probability with cells. For particles to induce pulmonary inflammation, they must deposit in the alveolar region. The deposition of (nano)particles depends mostly on their (agglomerate) size. Nanoparticles with a primary or agglomerate particle size between 10 and 100 nm will deposit more efficiently in the alveolar region compared to particles with an agglomerate particle size between 0.1 and 1 μm [16-20]. At a similar mass based exposure dose, particles of different sizes will have a different deposition pattern in the lungs, and the deposited dose in the alveoli ultimately determines the extent of the pulmonary toxic effects. The previous studies did not link the deposited dose in the alveoli to the observed effects [10-15].

Until now, the formulation in which silver nanoparticles induce toxicity remains unclear. The effects might be caused by the silver nanoparticles itself, the released silver ions, or a combination of both. Next to this, it remains unclear to what extend the geometric size of silver particles affect the induction of pulmonary inflammation. Since particle size is the most important particle characteristic that determines the deposited dose in the lungs and is of influence on the dissolution rate of silver nanoparticles, the aim of this study is to investigate the influence of particle size on pulmonary toxicity of silver nanoparticles. We hypothesize that small silver nanoparticles will induce more prominent pulmonary toxicity compared to larger silver particles because of the larger deposited dose in the alveoli and the higher dissolution rate. In the present study, we tested the effects of a similar mass exposure concentration of 15 nm and 410 nm silver (nano)particles after short-term nose-only inhalation exposure. The total lung burden was measured and used together with the exposure measurements as an input for the multiple path particle dosimetry (MPPD) model to estimate the alveolar dose. Transmission electron microscopy (TEM) was used to localize silver particles in the lung tissue and tissue distribution was measured to determine any differences in the kinetics of the silver particles. To determine the toxicity induced by 15 nm and 410 nm silver (nano)particles, body weight, total blood cell counts, inflammatory and cell damage markers in the bronchoalveolar lavage fluid (BALF) and histology of the lungs were analysed. All endpoints were determined at 24 hours and 7 days after the last exposure to investigate the possible recovery of adverse effects.

## Results

### Nanoparticle characteristics

**Figure 1:**
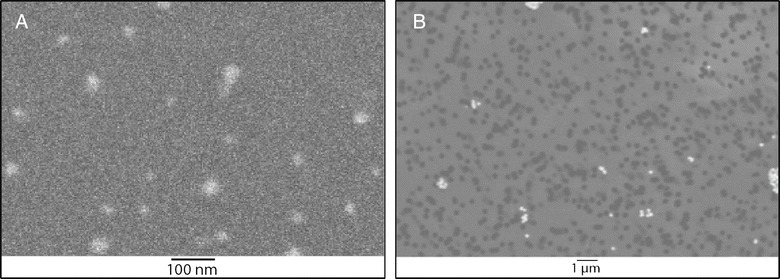
**Scanning electron microscopy (SEM) pictures of the 15 nm silver nanoparticles (A) and the 410 nm silver particles (B) captured on polycarbonate filters with 0.22 μm pores (Pictures are made in back-scatter mode).**

The particle characteristics are summarized in Table 1 and shown in Additional file 1: Figure S1. The silver nanoparticles had a count median diameter of 15 nm (Figure 1) and the exposed dosage was 179 μg/m^3^ air. For the larger silver particles, the 200 nm primary silver particles agglomerated to a count median diameter of 410 nm (Figure 1) and the exposed dosage was 167 μg/m^3^ air. While the mass dosage was similar for both particle groups, the particle number dosage was 3.8 × 10^6^ particles per cm^3^ for the 15 nm nanoparticles and 2.0 × 10^4^ particles per cm^3^ for the 410 nm particles.

**Table 1:** **Nanoparticle characteristics**

**Agglomerate size [nm]**	**Mass [μg/m** ^**3**^ **]**	**Particle number [#/cm** ^**3**^ **]**	**Surface area [nm** ^**2**^ **/cm** ^**3**^ **]**
15.3 (1.26)	179 (107–252)	3.8 × 10^6^ (2.6 - 5.0)	6.72 × 10^9^ (3.97-9.49)
410 (1.43)	167 (147–187)	2.0 × 10^4^ (0.43 - 3.7)	2.33 × 10^8^ (0.28-2.61)

Particle size is given as count median diameter (CMD) with the geometric standard deviation (GSD). Mass, particle number and surface area shown as mean with a 95% confidence interval.

### Experimental design

Since male rats showed more sensitivity towards induction of pulmonary inflammation compared to female rats after 90 days inhalation exposure [13,14], we chose to expose male rats. The rats were nose-only exposed for 6 hours per day, 4 consecutive days to fresh air, 15 nm silver nanoparticles or 410 nm silver particles, 12 rats per exposure group. Of each exposure group, 6 rats were sacrificed 24 hours after exposure and the other 6 rats were sacrificed 7 days after exposure.

### Quantification of silver in tissues by high resolution inductively coupled plasma mass spectrometry (HR-ICP-MS)

**Figure 2:**
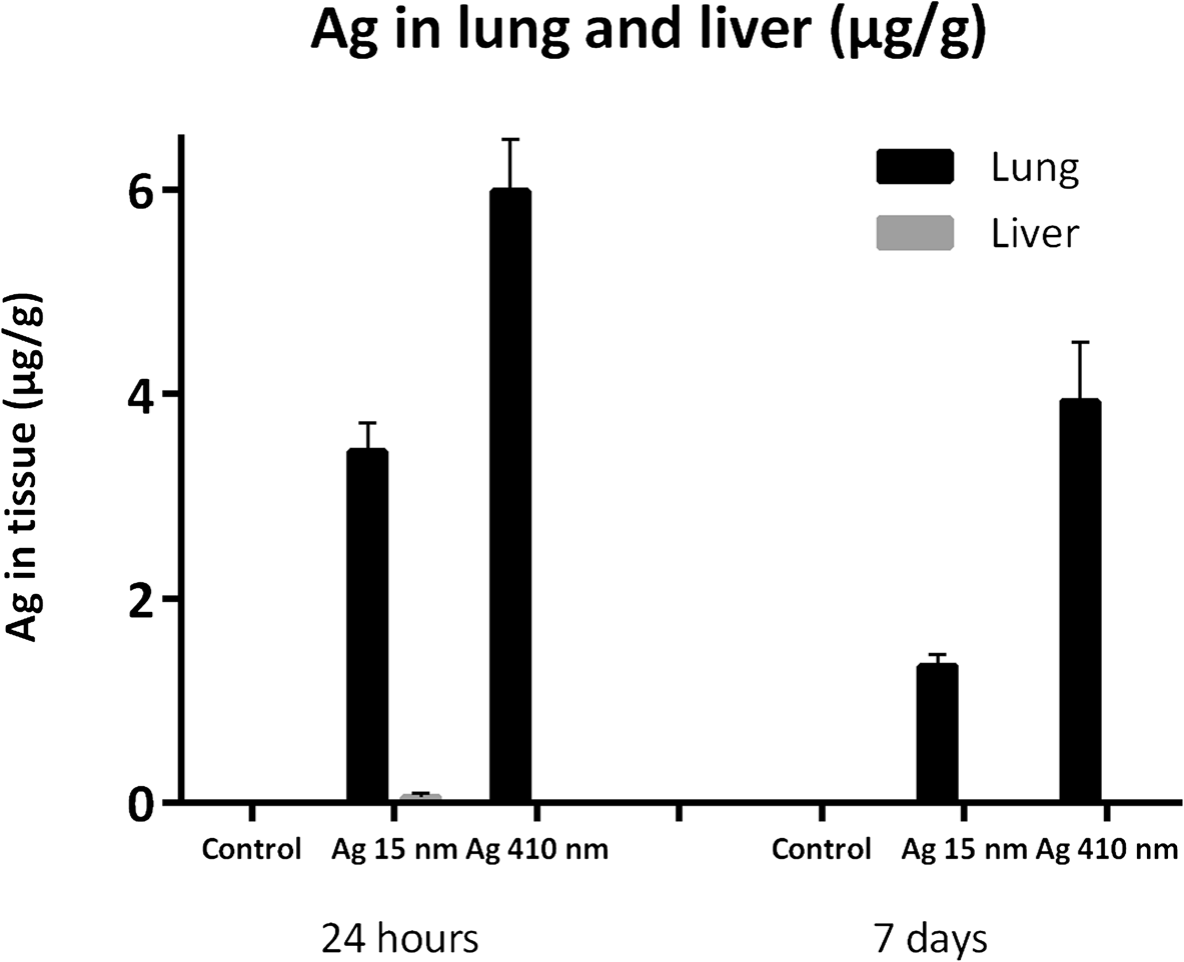
**Amount of silver detected in the lungs and liver by HR-ICP-MS at 24 hours and 7 days after exposure.** In the liver, silver could be detected in the animals exposed to 15 nm silver nanoparticles and not in the animals exposed to 410 nm silver particles. For both particle sizes, the amount of silver in the lungs at 7 days after exposure was significantly lower compared to 24 hours after exposure.

The presence of silver was measured with HR-ICP-MS in the lungs, liver, spleen, kidneys, brain, testis and lung-associated lymph nodes. Of these tissues, silver was detected in the lungs and liver (Figure 2). At 24 hours after exposure, the lungs of the rats exposed to 15 nm nanoparticles contained 3.4 μg silver per gram lung, whereas the lungs of the rats exposed to 410 nm particles contained 6.0 μg silver per gram lung. This equals a total lung deposit (TLD) of 5.5 μg of the 15 nm and 8.5 μg of the 410 nm silver particles, respectively. Because the silver nanoparticles are spherical, we could use these measurements to estimate the number of silver nanoparticles deposited in the lungs. A total lung capacity (TLC) of 13.7 ml [16] was used that results in 2.0 × 10^7^ particles per mm^3^ of 15 nm nanoparticles and 1.6 × 10^3^ particles per mm^3^ of 410 nm particles in the lungs. In the lung-associated lymph nodes, silver could be detected in one animal exposed to 15 nm silver nanoparticles. In the other animals, the level of silver in the lymph nodes was below the detection limit. In addition, the level of silver in the other tested tissues (spleen, kidneys, testis, brain) was below the detection limit of 0.01 μg/g tissue. At 7 days after exposure, the silver content in the lungs decreased to 1.3 μg/g tissue (2.1 μg TLD) for the rats exposed to 15 nm silver nanoparticles and 3.9 μg/g tissue (5.9 μg TLD) for the rats exposed to 410 nm silver particles. In the liver, silver was detected in the rats exposed to 15 nm silver nanoparticles at a level of 0.06 μg/g tissue (0.53 μg total) at 24 hours after exposure and 0.01 μg/g tissue (0.095 μg total) at 7 days after exposure, indicating removal of silver. No silver was detected in the liver of rats exposed to 410 nm silver particles at either time points.

### Estimated deposited dose in lungs using Multiple Path Particle Dosimetry Model (MPPD model)

According to the MPPD model, the total deposited fraction in the lungs is similar for both particle sizes [16] at 0.69 for the 15 nm nanoparticles, and 0.67 for the 410 nm particles. However, according to our HR-ICP-MS measurements, the TLD was 5.5 μg for the 15 nm nanoparticles and 8.5 μg for the 410 nm particles at 24 hours after exposure. The lower silver content in the lungs of the animals exposed to 15 nm particles might be explained by clearance of silver in the time between the end of the exposure and the sacrifice and after each day of exposure. The deposition in the alveoli differs between the two particle sizes: the deposited fraction in the alveoli is 0.27 for the 15 nm nanoparticles and 0.048 for the 410 nm particles [16]. Based on the HR-ICP-MS results, we estimated the deposited mass in the alveolar region was 2.1 μg for the 15 nm nanoparticles and 0.6 μg for the 410 nm particles at 24 hours after exposure, which equals 7.9 × 10^6^ particles per mm^3^ for the 15 nm nanoparticles and 118 particles per mm^3^ for the 410 nm particles. Based on mass, the deposited dose in the alveoli was 3.5 times higher in the animals exposed to 15 nm silver nanoparticles compared to the animals exposed to 410 nm silver particles at 24 hours after exposure. Based on particle number, the deposited dose in the alveoli was 66,000 times higher for the 15 nm compared to the 410 nm particle group.

### Electron microscopy of the lung

As reported above, the estimated number of silver particles in the alveoli was 7.8 × 10^6^ particles per mm^3^ for the 15 nm nanoparticles and 118 particles per mm^3^ for the 410 nm particles. The ultrathin TEM coupes are 70 nm thick; therefore, the expected number of particles per mm^2^ is 8 for the 410 nm particles, which may be too few to be detected. In contrast, for the 15 nm nanoparticles, the expected number is much higher: 5.5 × 10^5^ nanoparticles per mm^2^. Despite of this relatively high number neither 15 nor 410 nm silver particles could be detected in TEM section of the in epon embedded lung tissue.

**Figure 3:**
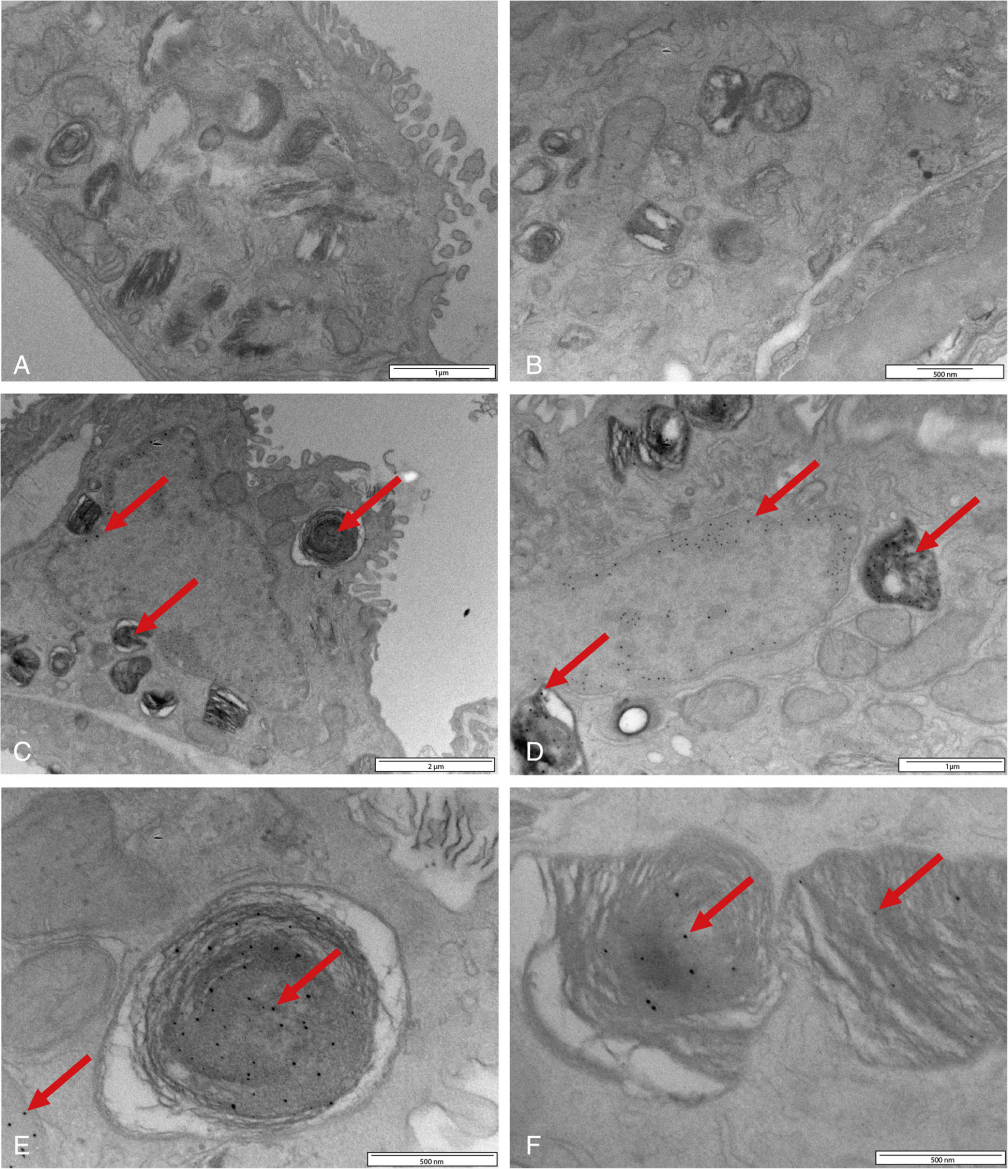
**TEM pictures of lung tissue of rats exposed to clean air (A and B) or 15 nm silver nanoparticles (C, D, E and F), afterwards followed by silver enhancement.** The pictures show the presence of silver nanoparticles in the nucleus, lamellar bodies and lysosomes of lung cells (indicated by the red arrows) at 24 hours after exposure.

Dissolution of silver nanoparticles is known to occur and will clearly result in a decrease in the size of the silver nanoparticles. Silver nanoparticles below 5 nm are difficult to detect in a biological sample by EM. Therefore, we employed the silver enhancement technique, a well-known approach to detect for instance 1 nm gold particles, normally not detectable in a biological sample by TEM. Silver enhancement also intensifies the EM signal of the silver nanoparticles by homogenous deposition of metallic silver on the particles surface, thereby increasing the particle size of the silver nanoparticles in the processed tissue. After silver enhancement on grids containing ultrathin lung sections, electron dense structures were detected by TEM in the lungs of the animals exposed to 15 nm silver nanoparticles (Figure 3), whereas they were almost absent in ultrathin lung sections of control animals. Two different grids containing ultrathin lung sections of control animals and three different grids containing ultrathin lung sections of animals exposed to 15 nm silver nanoparticles were examined for the presence of silver nanoparticles; at least 50 cell profiles per ultrathin section were examined. After silver enhancement, in the lung sections of the control animals silver dots were detected in 2% of the examined cells, whereas in the lung sections from the animals exposed to 15 nm silver nanoparticles silver dots were detected in 56% of the examined cells. The silver nanoparticles were both present in alveolar macrophages and lung epithelial cells (Figure 3). The nanoparticles were located mostly in vesicle-like structures and in the nucleus (Figure 3). However, in some cells the silver dots seemed randomly spread. The size of these deposits after silver enhancement was about 15 – 20 nm. This indicates that the original particle size of the silver nanoparticles in the lung tissue, without deposition of metallic silver, was smaller than 5 nm (P. van de Plas, Aurion, Wageningen, the Netherlands; personal communication).

### Clinical examinations

Exposure to silver nanoparticles of 15 nm and silver particles of 410 nm did not induce any premature mortality. There were no effects on animal attitude, fur, activity pattern, food and water consumption and faeces and urine production, and no effects on body weights and organ weights throughout the study (Table 2).

**Table 2:** **Animal weights given in grams**

**Group**	**Arrival**	**Start of exposure**	**24 hours after exposure**	**7 days after exposure**
Control	187 (178 – 197)	235 (218 – 253)	232 (220 – 243)	250 (237 – 263)
15 nm Ag	188 (179 – 197)	237 (228 – 245)	231 (222 – 240)	248 (243 – 253)
410 nm Ag	187 (177 – 197)	240 (228 – 253)	234 (221 – 247)	243 (229 – 258)

Data shown as mean with a 95% confidence interval.

### Hematology

**Figure 4:**
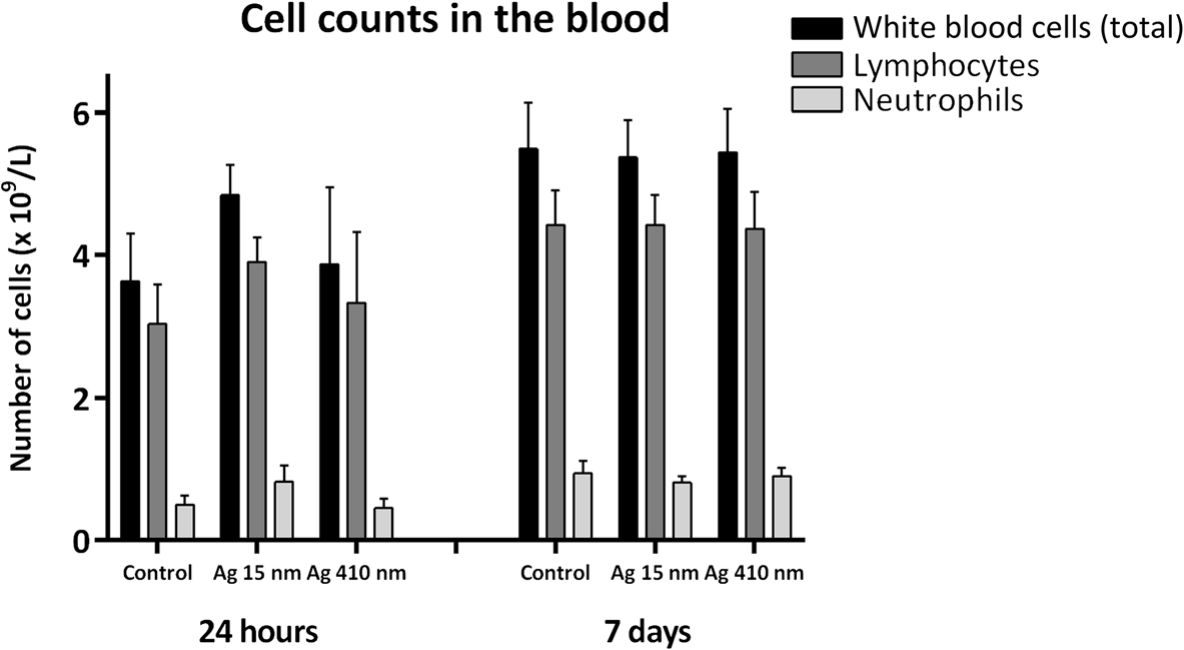
**Number and type of cells in the blood after exposure to 15 nm and 410 nm silver particles.** At 24 hours after exposure, the total amount of white blood cells was slightly increased in the group exposed to 15 nm silver nanoparticles compared to the controls as well as the number of neutrophils and lymphocytes. The differences were not statistically significant.

In the blood, the total amount of white blood cells and the amount of neutrophils and lymphocytes were all increased in the group exposed to the 15 nm silver nanoparticles compared to the other groups at 24 hours after exposure (Figure 4). However, the differences were not statistically significant. At 7 days after exposure, there was no increase in any of the measured blood cells. There were no increases in inflammatory blood cells in the group exposed to 410 nm silver particles at both time points. The level of Fibrinogen in the plasma did not differ between the controls and the exposed groups at both time points (data not shown).

### Histopathology

The histopathology analysis showed no differences between the groups. No lesions were observed in any of the lungs (results not shown).

### Total cell counts and differential cell counts in the bronchoalveolar lavage fluid (BALF)

**Figure 5:**
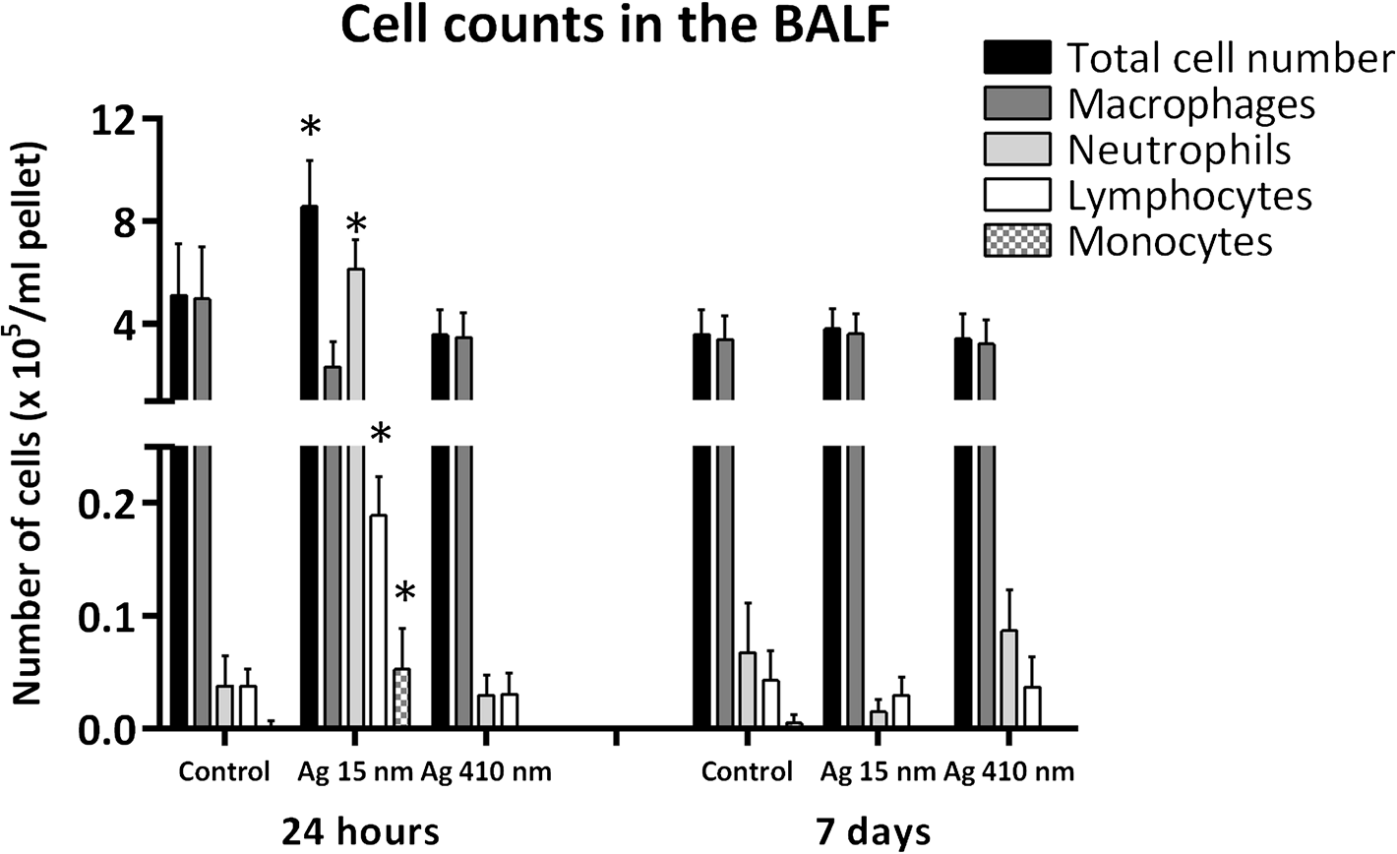
**Cell counts in the BALF of rats.** The group exposed to 15 nm silver particles had an increased number of total cells (*p =* 0.06), neutrophils (*p* < 0.01), lymphocytes (*p* < 0.05) and monocytes (*p* < 0.01) compared to the control group at 24 hours after exposure. Compared to the group exposed to 410 nm silver particles, the increased numbers of total cells (*p* < 0.05), neutrophils (*p* < 0.01), lymphocytes (*p* < 0.05) and monocytes (*p* < 0.001) were also significant. The increased cell numbers returned to normal at 7 days after exposure.

**Figure 6:**
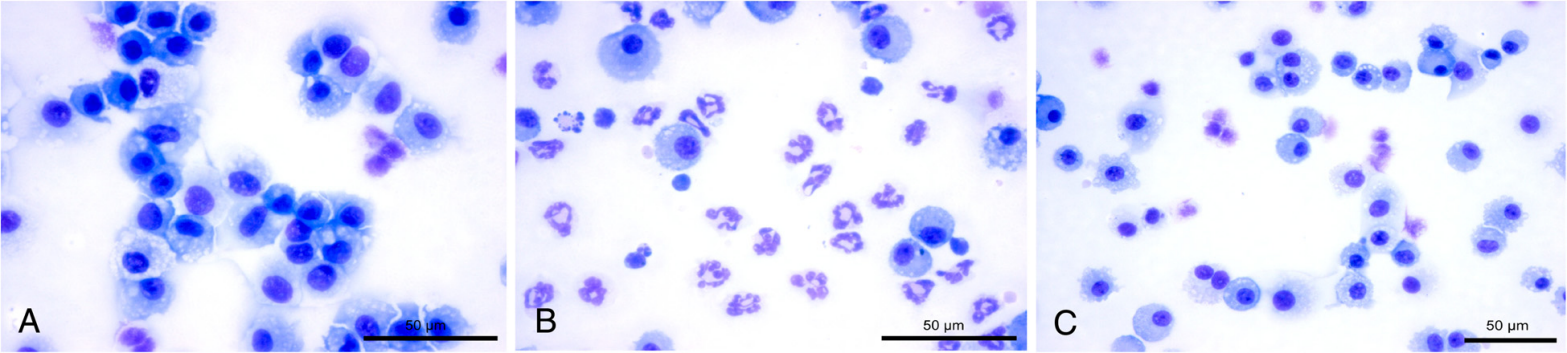
**Light microscopic images of cells in the broncho-alveolar lavage fluid of the rats at 24 hours after exposure. A**. Animal exposed to clean air. **B**. Animals exposed to 15 nm silver nanoparticles. **C**. Animals exposed to 410 nm silver particles. There is a significant influx of neutrophils in the lung fluid of the animals exposed to 15 nm silver nanoparticles.

The cell counts in the bronchoalveolar lavage fluid (BALF) are shown in Figure 5. The total cell number was increased 24 hours after exposure to 15 nm silver nanoparticles compared to control (*p* = 0.063) and significantly increased compared to the group exposed to 410 nm silver particles (*p* < 0.05). The increased cell number is caused by a neutrophil influx (Figure 6). 24 hours after exposure, there was an average of 3.95 × 10^5^ neutrophils per ml cell pellet in the group exposed to 15 nm silver nanoparticles compared to 2.3 × 10^3^ neutrophils per ml pellet in the controls (*p* <0.01) and 2.1 × 10^3^ neutrophils per ml pellet in the 410 nm silver particles exposed animals (*p* < 0.01). In addition, the number of lymphocytes in the BALF was increased after exposure to 15 nm silver nanoparticles (*p* < 0.05 compared to control, *p* <0.05 compared to 410 nm silver). The number of monocytes in the BALF 24 hours after exposure was also increased: 3300 monocytes per ml pellet in the group exposed to 15 nm silver nanoparticles compared to 150 in the controls and 0 in the group exposed to 410 nm silver particles (p < 0.01 and p < 0.001). Remarkably, the number of macrophages in the BALF was decreased 24 hours after exposure to 15 nm silver nanoparticles compared to control, although not statistically significant. There were no differences between the groups in number of multi-nuclei macrophages. There were no eosinophils detected in any of the exposed groups 24 hours after exposure.

The cell counts in the BALF at 7 days after exposure were completely different from those at 24 hours after exposure. At 7 days after exposure, most observed effects disappeared. There were no differences in the number of lymphocytes, macrophages and monocytes between the exposure groups. There was a slight decrease in the number of neutrophils in the BALF of rats exposed to 15 nm silver nanoparticles compared to both control and 410 nm silver particles, although not statistically significant.

For the groups exposed to 15 nm silver nanoparticles, the cell counts were significantly different between 24 hours and 7 days after exposure, for all cell types. Compared to 24 hours after exposure, the total cell number, neutrophils, lymphocytes and monocytes all significantly decreased and the number of macrophages, multi-nuclei macrophages significantly increased at 7 days after exposure.

### LDH and total protein in the BALF

**Figure 7:**
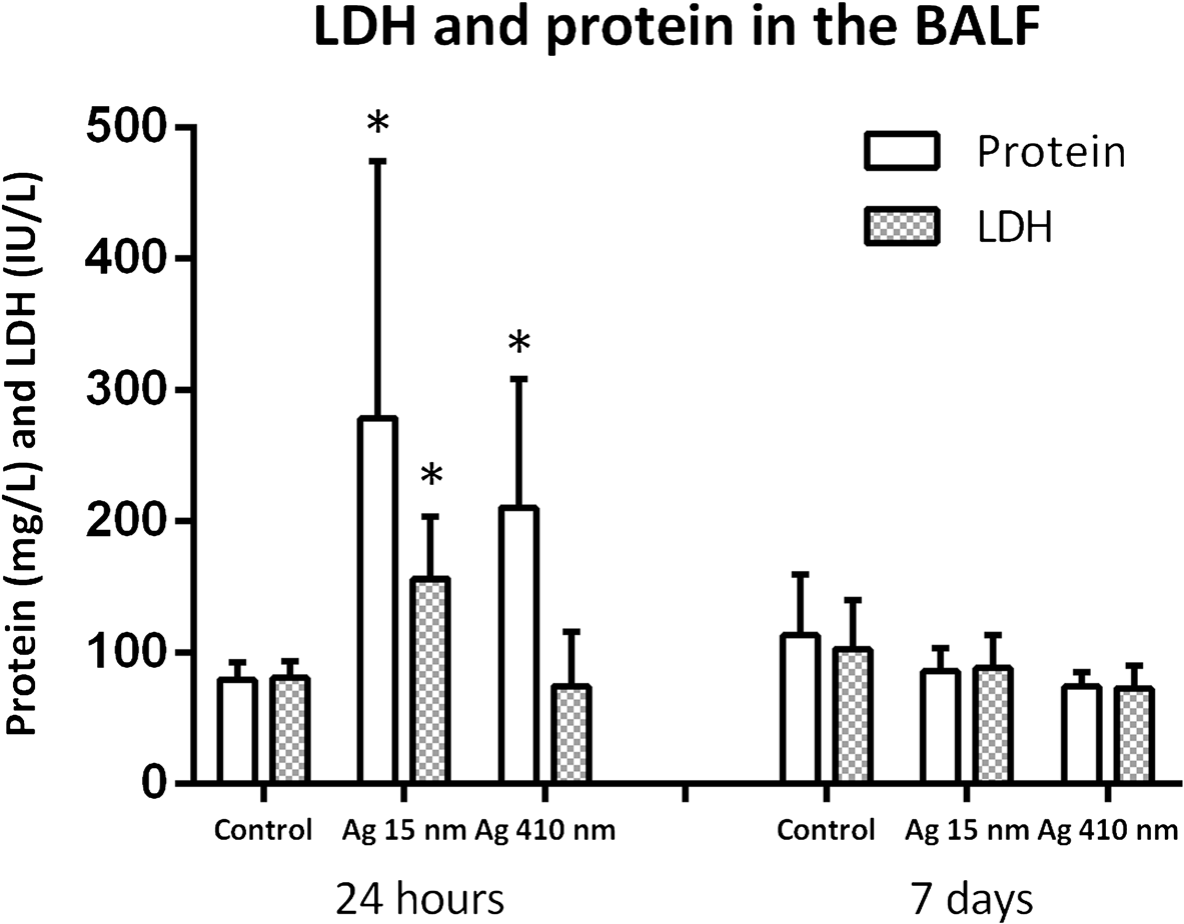
**The level of total protein and LDH in the supernatant of the broncho-alveolar lavage fluid.** At 24 hours after exposure, both the animals exposed to 15 nm silver particles and 410 nm silver particles showed a significant increase in the level of total protein compared to control (*p* < 0.01 and p < 0.05, respectively). The total protein levels returned to normal at 7 days after exposure. The level of LDH was also significantly increased in the animals exposed to 15 nm silver nanoparticles at 24 hours after exposure compared to the control (*p* < 0.05) and to the animals exposed to 410 nm silver particles (*p* < 0.05).

The supernatant of the BALF was used to determine lactate dehydrogenase (LDH) release and total protein as markers for cell damage (Figure 7). At 24 hours after exposure, the groups exposed to 15 nm silver nanoparticles and 410 nm silver particles both showed significantly increased levels of total protein in the BALF compared to the controls (*p* < 0.01 and p < 0.05 respectively). The level of LDH was doubled at 24 hours after exposure to 15 nm silver nanoparticles compared to both control (*p =* 0.05) and compared to 410 nm silver particles exposed animals (*p* < 0.05). At 7 days after exposure, the levels of both total protein and LDH in the BALF of the exposed animals decreased to control levels.

### Pro-inflammatory cytokines in the BALF

**Figure 8:**
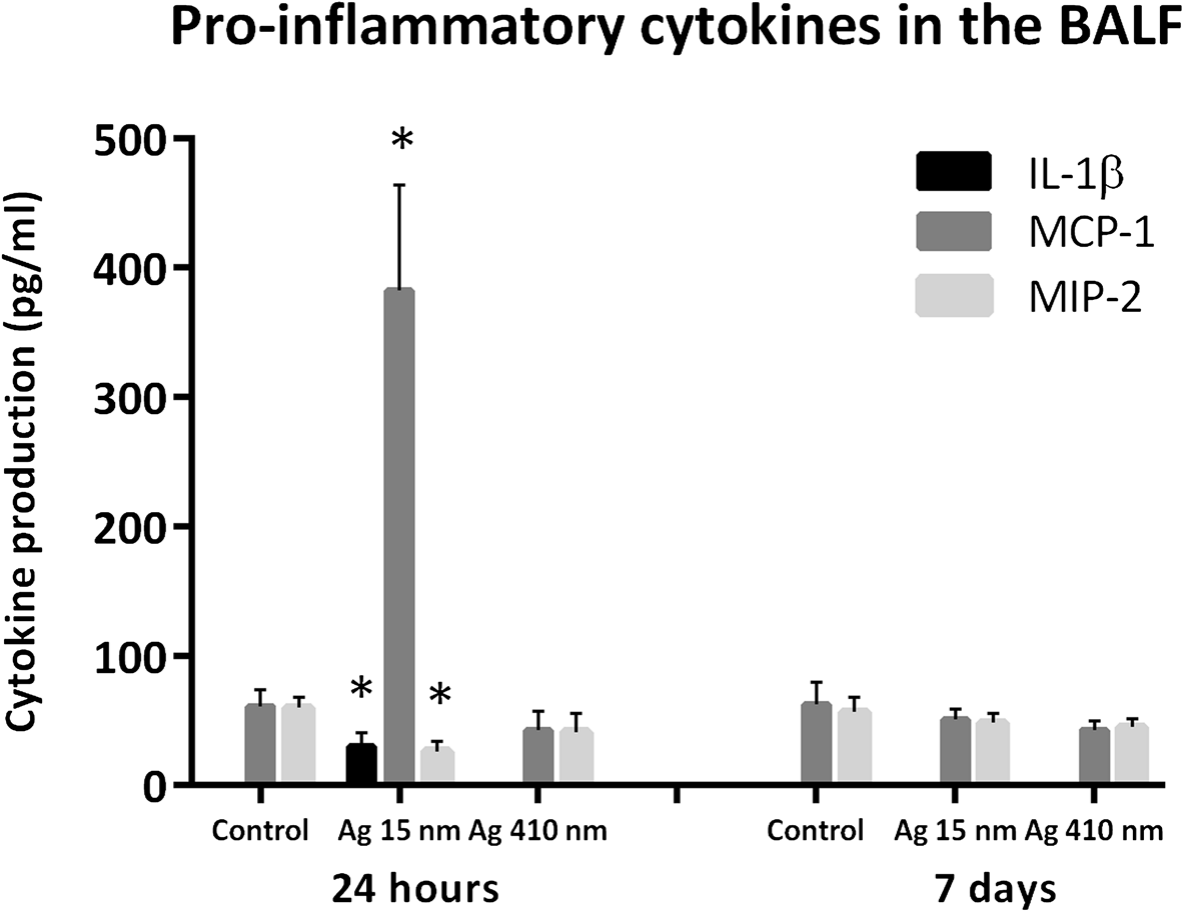
**Cytokine level in the broncho-alveolar lavage fluid.** The animals exposed to 15 nm silver nanoparticles had significantly increased levels of IL-1β (*p* < 0.05), MCP-1 (*p =* 0.06) and MIP-2 (*p* < 0.01) in the BALF at 24 hours exposure compared to the controls. At 24 hours after exposure, the levels of IL-1β (*p* < 0.05) and MCP-1 (*p* < 0.01) were also significantly increased compared to the animals exposed to 410 nm silver particles. At 7 days after exposure, the cytokine levels returned to normal.

The supernatant of the BALF was used to determine the level of pro-inflammatory cytokines. 12 different pro-inflammatory cytokines were selected: IL-1β, IL-6, TNF-α, IFN-γ, IL-13, GM-CSF, MCP-1, IL-12p70, IL-18, MIP-1a, MIP-2 and RANTES. Of these cytokines, only IL-1β, MCP-1 and MIP-2 could be measured; all the other cytokines were and stayed below or around the detection level (Figure 8). Both IL-1β and MCP-1 were significantly increased 24 hours after exposure to 15 nm silver nanoparticles. IL-1β was detected at 30.2 pg/ml for the 15 nm nanoparticles exposed group compared to 5.5 pg/ml for the controls (*p* < 0.05) and 4.9 pg/ml for the 410 nm particles exposed group (*p* < 0.05). In addition, the level of MCP-1 was increased in the rats exposed to 15 nm nanoparticles at 24 hours after exposure: 382.7 pg/ml compared to 61.6 pg/ml of the controls (*p =* 0.063) and 42.8 in rats exposed to 410 nm silver particles (*p* < 0.01). The level of MIP-2 was significantly decreased to 26.5 pg/ml after 24 hours after exposure to 15 nm silver nanoparticles compared to the level of 60.2 pg/ml in the controls (*p* < 0.01). The changes in IL-1β, MCP-1 and MIP-2 levels at 24 hours after exposure to 15 nm silver nanoparticles recovered at 7 days after exposure, when no significant differences were observed in any of the cytokine levels measured between the different exposure groups. There were no significant differences between the group exposed to 410 nm silver particles and the controls at both 24 hours and 7 days after exposure.

### Oxidative stress in the lungs

**Figure 9:**
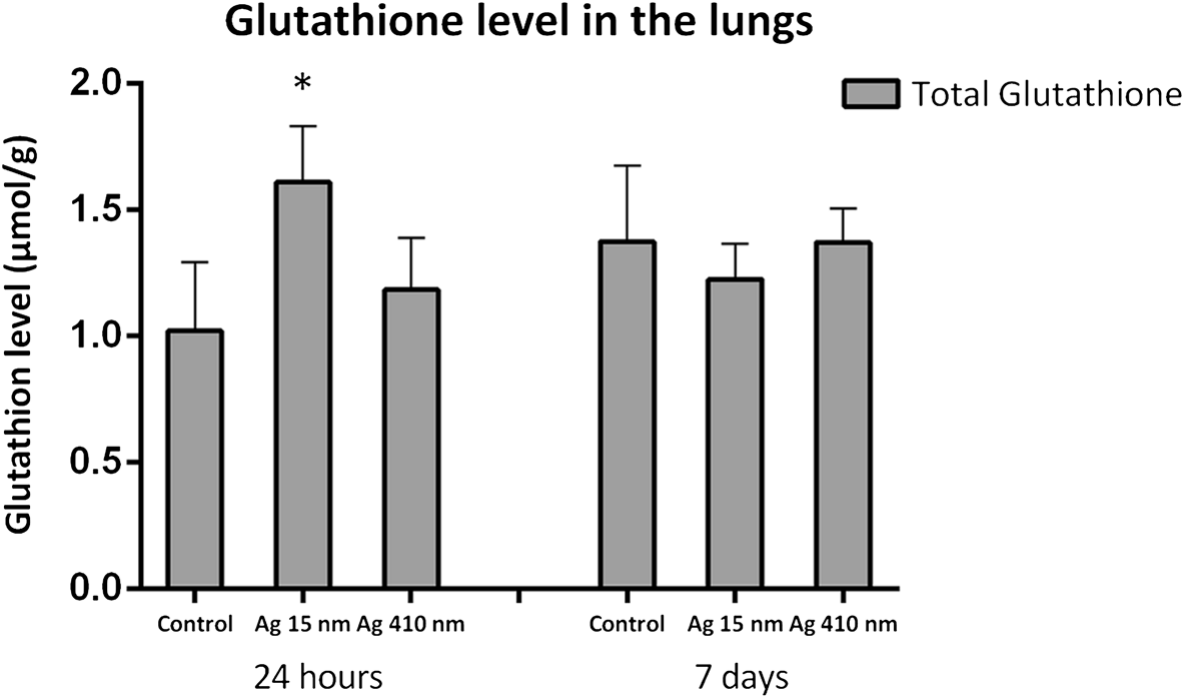
**Glutathione production in the lungs of rats.** At 24 hours after exposure, the total glutathione production in the 15 nm silver nanoparticles exposed group was significantly elevated compared to the controls (*p* < 0.01) and the 410 nm silver particles exposed group (*p* < 0.05).

The levels of total glutathione, oxidized (GSSG) and reduced (GSH) glutathione were measured in the homogenate of the rinsed right lung of all the rats (Figure 9). The level of total glutathione was significantly increased at 24 hours after exposure to 15 nm silver nanoparticles compared to the controls (*p* < 0.01) and compared to the group exposed to 410 nm silver particles (*p* < 0.05). The level of reduced glutathione was also significantly increased at 24 hours after exposure to 15 nm silver nanoparticles compared to the controls (*p* < 0.01) and the 410 nm exposed group (*p* < 0.05), whereas the level of oxidized glutathione was not. The ratio of GSH/GSSG was not significantly different between any of the exposure groups. 7 days after exposure, the level of total glutathione and reduced glutathione in the group exposed to 15 nm silver nanoparticles returned to control levels.

## Discussion

In this short-term inhalation study, silver nanoparticles of two different sizes were tested to determine the differences in the lung deposition, tissue distribution and adverse effects. Exposure to 15 nm silver nanoparticles induced a moderate inflammatory response at 24 hours after the end of the exposure period. All the observed effects disappeared at 7 days after exposure. The most pronounced observed effect was the influx of neutrophils at 24 hours after exposure to 15 nm silver nanoparticles, which was about 175 times larger compared to the controls. In addition, we found release of pro-inflammatory cytokines, an increase in total glutathione and the presence of silver nanoparticles inside lung cells. In contrast, the 410 nm particles induced none of these effects in the lung, clearly demonstrating a particle size dependent difference in the induction of pulmonary inflammation.

There are two main reasons to explain the noted differences in effects by the two silver particles. First, the differences in effects may be attributed to the differences in lung deposition of the smaller versus the larger silver particles. The total lung deposit as measured by HR-ICP-MS was 5.5 μg for the 15 nm silver nanoparticles and 8.5 μg for the 410 nm silver particles at 24 hours after the end of the exposure period. According to the MPPD model, the fraction that reaches the alveoli is about 5.5 times higher for the 15 nm nanoparticles compared to the 410 nm particles. Based on the HR-ICP-MS results we estimated the amount of 15 nm silver nanoparticles present in the alveoli was 7.9 × 10^6^ particles per mm^3^ (2.1 μg mass or 5.8 × 10^12^ nm^2^/cm^3^ surface area) and the number of 410 nm silver particles was 118 particles per mm^3^ (0.6 μg mass or 6.2 × 10^10^ nm^2^/cm^3^ surface area). Therefore, the number of bioavailable 15 nm nanoparticles in the alveoli is about 66,000 times larger compared to the 410 nm particles, resulting in a higher interaction probability with cells.

Comparing same mass deposits of microparticles and nanoparticles, the latter contains a much higher number of particles that need to be cleared. In addition, the smaller silver nanoparticles deposit deeper in the lungs where they are less efficiently cleared compared to the larger particles that deposit more in the conducting airways where they are more easily cleared [16-18,21-24]. Moreover, the phagocytosis of nanoparticles by macrophages is less efficient [25-27] and slower compared to microparticles [28-30]. Our results, however, show a clearance from the lungs of 62% of the 15 nm nanoparticles compared to 31% of the 410 nm particles at 7 days after exposure. The higher clearance rate of the smaller particles might be explained by translocation from the lungs of the smaller silver nanoparticles or the released ions to the blood or interstitium. This is further supported by the detection of silver in the liver of animals at 24 hours after exposure to 15 nm silver nanoparticles, indicating systemic availability of silver particles or ions. This was not detected after exposure to the 410 nm silver particles.

Previous inhalation studies with nanosilver showed a dose-dependent increase in silver concentration in the liver [11,13,15] and even a low translocation to the brain and olfactory bulb [11,15]. In one study, the amount of silver in the brain and olfactory bulb was 2.2 ng/g and 6.97 ng/g, respectively, after exposure to the highest dose tested of 61 μg/m^3^ [11]. In the other study, the amount of silver in the brain and olfactory bulb was 18.63 ng/g and 30.48 ng/g at the highest dose tested of 515 μg/m^3^ [15]. In both studies, the tissue distribution of silver was detected by atomic absorption spectrophotometer. The reported level of silver is too low to detect with the HR-ICP-MS, which has a detection limit of 0.01 μg/g, possibly explaining why we did not detect any silver in the brain or olfactory bulb. It should be noted that both detection methods do not distinguish between silver particles and silver ions.

The second reason for the observed differences in effects between the 15 nm and 410 nm silver particles is the difference in particle dissolution. It is known that smaller particles dissolve faster compared to larger particles because of the high surface to volume ratio [31]. Several *in vitro* studies determined the dissolution rate of silver nanoparticles. In the study of Ma et al. [31], the dissolution of silver nanoparticles ranged from 1% for the larger particles of 80 nm to 60% for the smallest particles of 5 nm at pH 8 during three and two months incubation, respectively. However, there was no constant release of silver ions per unit of surface area, indicating that surface area alone did not explain the dissolution of silver nanoparticles [31]. According to this and other studies [31-35], the dissolution of silver nanoparticles depends on their particle size, the pH of the solution, the ions present in the solution (e.g. sulphate, chloride and phosphate causing precipitations with silver ions or catalysing dissolution), aggregation state and the incubation time. Giving these *in vitro* results, the dissolution rate of the 15 nm silver nanoparticles will probably be higher compared to the 410 nm silver particles resulting in an increased ion release. None of these studies report complete dissolution of silver nanoparticles, the effects observed after exposure to silver nanoparticles can be induced by the released ions, the silver nanoparticles itself or a combination of both.

In a study of Pratsinis et al. [36], the toxicity of silver nanoparticles in the presence of their released ions, and the released ions alone was tested *in vitro*. For small silver nanoparticles with a size below 10 nm, the released silver ions from the surface dominated the cytotoxicity whereas for larger silver nanoparticles the interactions with silver particles dominated the toxicity [36]. According to Beer et al. [37], at silver ion fractions ≤2.6% the silver nanoparticles contribute to the observed toxicity while at silver ion fractions ≥5.5% the silver nanoparticles do not add additional toxicity *in vitro* [37]. In a recent published study [38], oropharyngeal aspiration in mice of 20 nm citrate-coated silver nanoparticles caused pulmonary inflammation indicated by neutrophil influx and the release of pro-inflammatory cytokines, whereas 110 nm citrate-coated silver particles caused a smaller neutrophil influx, only at the highest dose tested, and no release of pro-inflammatory cytokines. At 21 days after exposure, the silver content in the lungs did not differ between 20 nm and 110 nm silver particles. In addition, for both particle sizes, there was no silver detected in any of the other selected organs and tissues. Solubility test showed that about 4% of the 20 nm nanoparticles and 2% of the 110 nm particles dissolved in 24 hours. Therefore, the authors concluded the larger pulmonary inflammation caused by the 20 nm silver nanoparticles is due to the higher bioavailability of silver ions. However, the effects of 20 nm silver nanoparticles were stronger compared to the effects of silver nitrate indicating the particles itself contributed to the observed effects. In contrast to these results, the 110 nm silver particles caused mild sub-chronic effects in the lungs at 21 days after exposure, which might be caused by a slower and more persistent silver ions release [38]. Similar to these studies, it is likely that the release of silver ions contributed to the observed effects after exposure to 15 nm silver nanoparticles in the present study. To further elucidate the mechanism of the silver nanoparticle toxicity, the dissolution behaviour of silver nanoparticles inside cells should be investigated in detail.

In our study, we could detect by TEM silver nanoparticles in the lung tissue of rats at 24 hours after exposure to 15 nm silver nanoparticles. At 24 hours after exposure to 410 nm silver particles, we could not detect silver particles in the lungs because the number of particles was too low. The detected silver nanoparticles had a particle size of 15–20 nm after silver enhancement, indicating their size in the lung tissue was smaller than 5 nm before enhancement. In combination with the literature on the dissolution of silver nanoparticles, these EM results are an indication that the silver nanoparticles probably partially dissolved after deposition resulting in a decrease in particle size and the release of silver ions. The dissolution might occur extra-cellular in the lung lining fluid and intra-cellular in vesicles in epithelial cells or macrophages. Besides the release of silver ions, we believe the silver nanoparticles itself may also have contributed to the observed effects because the TEM images show the presence of silver nanoparticles inside lung cells. The 15 nm silver nanoparticles in our study dissolved over time resulting on the one hand in the release of toxic silver ions and on the other hand in a decrease in particle size to <5 nm which gives the particles the opportunity to pass the cellular membranes and the nuclear pores. Previous studies report that particles smaller than 5 nm possibly can pass the nuclear pores [39], while particles of 20 nm do not reach the nucleus and could be detected in vesicles and lamellar bodies *in vitro* [40]. Once inside the cells and eventually inside the nucleus, silver nanoparticles are a continued source of ion release, leading to more damage compared to silver ions released from silver nitrate [41]. The intracellular ion release has been reported previously as the Trojan-horse type mechanism [42,43]; nanoparticles can more easily enter the cells compared to the ions itself. The higher dissolution rate, in combination with the higher alveolar deposition of the 15 nm nanoparticles, might explain why the 15 nm nanoparticles caused toxicity and the 410 nm particles did not. Based on the results of the present study, it is not possible to quantify how much of the observed effects can be attributed to the difference in alveolar deposition and how much of the effects can be attributed to cellular uptake and ion release.

Findings of particle size related pulmonary inflammation are not limited to silver nanoparticles. Other inhalation studies with titanium dioxide, carbonaceous, nickel oxide, zinc oxide, ferric oxide, aluminium oxyhydroxide, quartz and cerium oxide particles also focused on the influence of particle size on the induction of pulmonary inflammation [27,44-52]. Indeed, several studies report that smaller nanoparticles caused a greater inflammatory response compared to larger particles when the same mass dosage was administered [27,44-48]. However, although particle size will affect the site and amount of deposited material, it seems that the local dose alone cannot explain the differences in toxicity. Solubility will at least be one of the other variables in an equation that would allow predicting adverse effects of nanoparticles after inhalation.

## Conclusion

The results of the present short-term inhalation study of uncoated silver particles indicate that particle size is an important characteristic that determines the induction of pulmonary inflammation. Exposure to 15 nm silver nanoparticles induced moderate pulmonary inflammation at 24 hours after exposure, whereas 410 nm silver particles did not. The lung deposition of the 410 nm silver particles was mainly in the upper airways; the number of particles deposited in the alveoli was calculated to be 66,000 times higher for the 15 nm silver nanoparticles. TEM analysis showed the presence of silver nanoparticles in lung cells that were decreased in particle size, demonstrating the *in vivo* dissolution of silver nanoparticles. Altogether, these findings strongly suggest that size-related silver nanoparticle induced pulmonary inflammation is a consequence of both size related lung deposition and dissolution rate.

## Methods

### Test material and characterization

**Figure 10:**
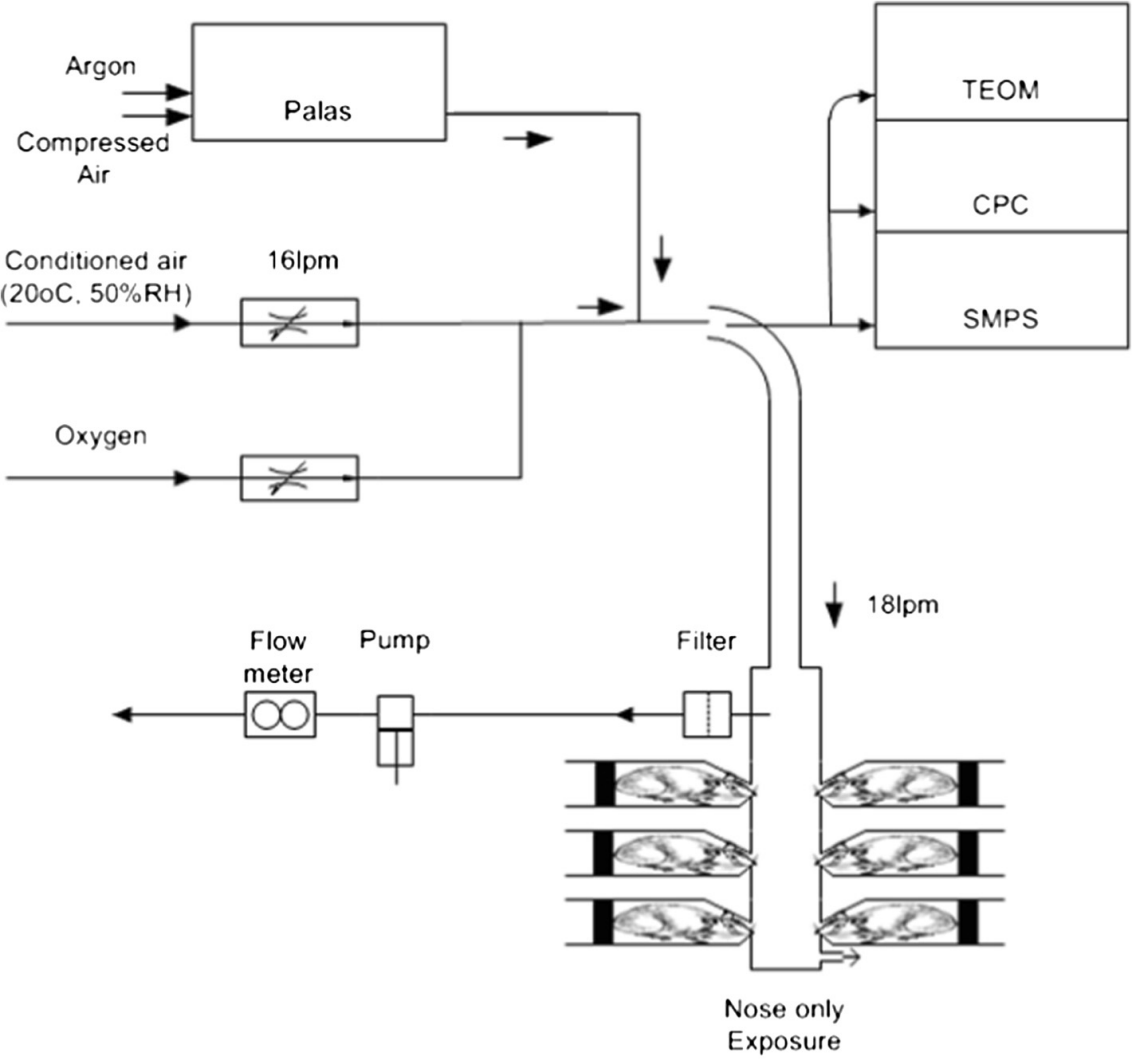
**Palas spark generator setup for the nose-only exposure to 15 nm silver nanoparticles.** The silver nanoparticles are generated by the Palas spark generator and subsequently mixed with conditioned air and oxygen. Particle size, number and mass are continuously monitored.

**Figure 11:**
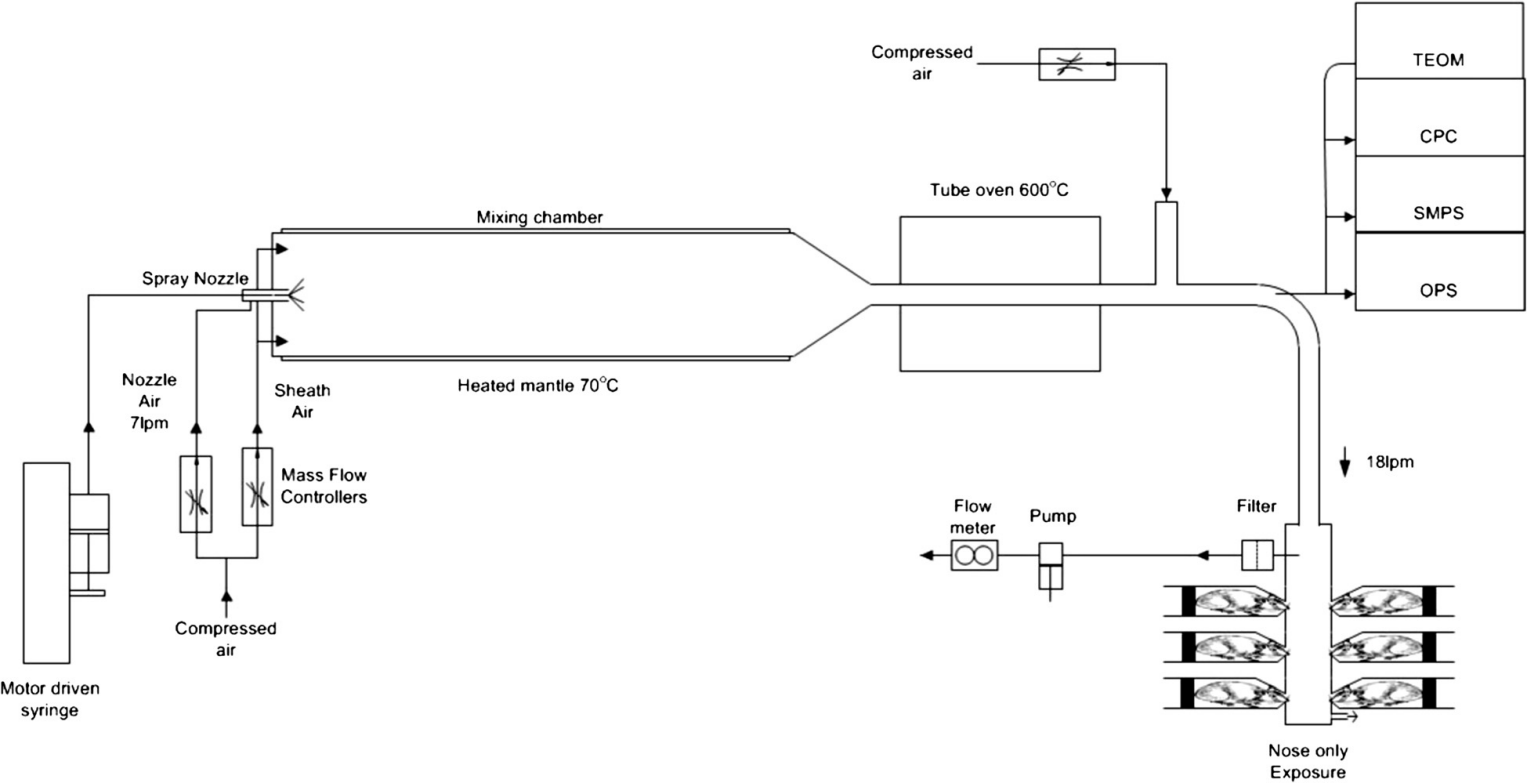
**Spray nozzle setup for the nose-only exposure to 410 nm silver particles.** The silver particles are in solution and sprayed into a heated mixing chamber. Subsequently, the particles are dried in an oven and mixed with compressed air. Particle size, number and mass are continuously monitored.

The test atmosphere was produced by mixing silver nanoparticles with HEPA filtered and conditioned (50% RH, 21°C) compressed air. Depending on the target particle size, one of two types of particle generators was used. The silver nanoparticles were produced by a Palas GFG 1000 (Palas GmbH, Karlsruhe, Germany) spark generator fitted with silver tipped copper electrodes. To generate the 15 nm nanoparticles the output of the generator was immediately diluted in conditioned air. Oxygen was added to the dilution air to compensate for the argon flow from the Palas, a final concentration of 20% oxygen in the airflow was maintained. The particle number concentration was controlled by setting the Palas spark frequency. The final condition of the aerosol (55%RH, 21°C) was set by adjusting the relative humidity of the mixing air (Figure 10). 200 nm silver particles were purchased by NanoComposix, inc. San Diego, USA. The 200 nm silver particles were polyvinylpyrrolidone (PVP)-coated and supplied in solution of 1 mg/ml in MilliQ water. The silver particle solution was forced through a small nozzle of a Schlick spray nozzle where it was broken up in small droplets (nebulized) by compressed air. The flow of the solution was controlled using a 20 ml Terumo syringe and a syringe pump (TSE model 540200, Bad homburg, Germany). The nebulized flow was mixed with compressed air in a heated mixing tube. After mixing and drying, the aerosol passed through a tube oven (700°C) to fuse the aggregates into a single particle. The heat of the oven removed the PVP-coating of the silver particles. After the oven, the aerosol diluted further with conditioned air and was allowed to cool. The mass concentration was controlled by adjusting the syringe pump motor speed and thus the flow of the solution. The final condition of the aerosol (55% RH, 21°C) was set by adjusting the relative humidity of the final mixing air (Figure 11).

Several measurements of the test atmosphere were performed during the exposure. In the main flow upstream of the nose only unit particle mass concentration, number concentration, and particle size distribution were measured. The particle mass concentration was determined by time aggregated gravimetric with Teflon R2PJ047 filter (Pall corp., Ann Arbor MI, USA) and by tapered element oscillating microbalance (TEOM) series1400 (Rupprecht & Patashnick, New York, USA). The particle number concentration was measured over time by a condensation particle counter (CPC) 3022 (TSI inc., St Paul MN, USA). Particle size distribution was monitored over time by an OPS 3330 (TSI inc., St Paul MN, USA), a scanning mobility particle sizer (SMPS) 3080 with 3085 Nano DMA (TSI inc., St Paul MN, USA) and a MOI Model No. 110 (MSP corp, Minneapolis MN, USA). Temperature and relative humidity were determined by a Vaisala M170 (Vaisala Oyj, Helsinki, Finland). The particle size was confirmed by scanning electron microscopy (SEM) using a Nova Nanolab 600 Dualbeam (FEI, Eindhoven, the Netherlands) (Figure 1). In addition, the gravimetric mass concentration was determined. A Sartorius MC-5 microbalance (Sartorius, Goettingen, Germany) was used in controlled relative humidity (40 – 45%) and temperature (21 – 23°C) conditions to do the mass measurements, the filters were weighed before and after each exposure. Laboratory and field blanks were used for quality assurance. The filter volume flow was measured with dry gas meters (Gallus 2000 G1.6, Actaris Meterfabriek B.V., Dordrecht, the Netherlands).

### Animals

The Animal Ethical Committee of the National Institute for Public Health and the Environment (Bilthoven, the Netherlands) approved the experimental design of the animal study at the 6th of July 2012. Healthy male Fischer rats (F344/DuCrl) were supplied by Charles River (Sulzfeld, Germany). The animals were kept under specific pathogen free (SPF) conditions. After an acclimatization period of two weeks, the animals were 10 weeks old at the start of the experiment. The rats were randomly allocated to the control and the test groups. During exposure, the animals were restrained in nose-only tubes (CH technologies, Westwood NJ, USA) fixed to the inhalation system. When not exposed, animals were housed up to 3 animals in macrolon III cages with filtertops to prevent dust entering the cages (bedding material: Lignocel S8-15, Altromin Spezialfutter GmbH & Co. KG). The animal room was air-conditioned with a 12 hours light- 12 hours dark cycle, the temperature ranging from 20 to 24°C with relative humidity ranging from 30 – 70%. Except during exposure, certified feed CRM (SDS Diets, UK) and water were available ad libitum. Before start of the exposure period, all animals were acclimatized to the nose only tubes on 3 consecutive days for 1 hour per day. At 24 hours and 7 days after the last exposure, rats were weighed, anesthetized by a single intraperitoneal injection of ketamine (75 mg/kg) and xylazine (10 mg/kg) and subsequently exsanguinated via the abdominal aorta.

### Experimental design

The rats were nose-only exposed for 6 hours per day, 4 consecutive days to fresh air, 15 nm silver nanoparticles or 410 nm silver nanoparticles, 12 rats per exposure group. Of each exposure group, 6 rats were sacrificed 24 hours after exposure and the other 6 rats were sacrificed 7 days after exposure. During nose-only inhalation, rats have a breathing pattern that results in a more realistic internal exposure than a single high dose in intratracheal instillation and oropharyngeal aspiration or possible additional oral exposure in whole-body exposure chambers.

### Quantification of silver in tissues by high resolution inductively coupled plasma mass spectrometry (HR-ICP-MS)

The presence of silver in the lungs, liver, spleen, kidneys, brain, testis and lung associated lymph nodes was determined by high resolution inductively coupled plasma mass spectrometer (HR-ICP-MS) [53,54]. Samples of homogenized tissue (max. 0.5 g) were completely dissolved on a block heater (Stuart SBH200D, supplied by Omnilabo, Breda, The Netherlands) for 24 hours at 120°C after the direct addition of aqua regia (0.5 mL 60% nitric acid, ultrapure, and 1.5 mL 30% hydrochloric acid, ultrapure) and afterwards diluted. The silver concentration in the digests was determined using high resolution inductively coupled plasma mass spectroscopy (HR-ICPMS; Element XR, Thermo Fisher Scientific, Bremen, Germany). Both silver isotopes (^107^Ag and ^109^Ag) were measured in the low-resolution mode. External calibration curves of the interference-free silver isotopes were used for quantification. On-line addition and correction with an internal standard (rhodium with the measured isotope ^103^Rh in the low-resolution mode) was applied too. The results of ^107^Ag were reported and the results of ^109^Ag were used for control. Sample pre-treatment and analysis were carried out in a cleanroom facility class 100 000.

### Estimated deposited dose in lungs using Multi Path Particle Dosimetry model (MPPD model)

To estimate the fractions of deposited dose in the head, tracheobronchial, and alveolar region of the rats, the multiple path particle dosimetry model (MPPD model) was used [16]. We used the default parameters of the model for rats, i.e., a forced respiratory capacity of 4 ml, head volume of 0.42 ml, nasal breathing, tidal volume of 2.1 ml, and a breathing frequency of 102/min. The inspiratory fraction was 0.5, and no pause was entered. Calculations were done using the count median diameter (CMD), geometric SD, the mass concentration, and a density of 10.49 g/cm^3^.

### Electron microscopy of the lung

Of two animals of each group, the left lung was cut out with the bronchus attached and fixed by pressure (2 kPa for 1 hour) with half-strength Karnovsky fixative (0.08 M Sodium-cacodylate buffer, 2.5% glutaraldehyde, 0.025 mM CaCl_2_, 0.05 mM MgCl_2_) for electron microscopy. After two days, the fixative was replaced with 2% para-formaldehyde in sodium-cacodylate buffer for storage. Of each lung, three slices of about 1 – 1.5 mm were cut out at the top, middle and bottom of the lung for further analysis. The tissue slices were post-fixed by osmium potassium ferro-cyanide solution (0.1 M sodium-cacodylate buffer, 1% osmium tetra-oxide, 1.5% potassium ferro-cyanide). Next, the tissue slices were dehydrated in aceton series, cut in smaller pieces and subsequently embedded in epon. The epon was polymerized at 60°C. The epon blocks were trimmed prior to sectioning. During sectioning, ultrathin sections of 70 nm were sectioned and put on 3 mm hexagonal copper grids coated with a formvarfilm. The sections were analysed by transmission electron microscopy (TEM) (Tecnai 10, 100 kV, FEI, Eindhoven, the Netherlands).

### Silver enhancement

A silver enhancement procedure was used to increase the particle size of the silver nanoparticles in the lung tissue to make it possible to detect the silver nanoparticles with electron microscopy. The silver enhancement reagents were purchased at Aurion and performed on grid according to manufacturer’s instructions (Wageningen, the Netherlands).

### Clinical examinations

The animals were examined three times on exposure days (before, during and after exposure) and once daily during the periods before and after exposure. The clinical examination included examination of attitude, animal fur, activity level, food and water intake, and faeces and urine production. The body weight of the animals was determined upon arrival, at the start of the exposure period and at the day of sacrifice.

### Hematology analysis

Blood samples were collected in EDTA-containing tubes and in citrate vials for analysis of cell types and inflammatory markers in the blood. The level of Fibrinogen in the citrate plasma was measured using a rat Fribrinogen ELISA kit (GenWay Biotech, Inc., San Diego, USA) according to manufacturer’s instructions.

### Histopathology

The lungs fixed with half-strength Karnovsky fixative were also used for histopathology. Of these lungs a thin slice was made over the length of the tissue, embedded in paraffin and stained by haematoxylin-eosin for histopathology analysis.

### Bronchoalveolar lavage fluid (BALF) analysis

The left lung of the rats was bound just below the bifurcation of the trachea and the right lung was cannulated via the trachea. Bronchoalvelar lavage was performed *in situ* by infusing the right lung three times with 27 ml/kg physiological saline. The retrieved bronchoalveolar lavage fluid (BALF) was kept on ice and centrifuged for 10 minutes at 400 g. The pellet was resuspended in physiological salt for analysis of the total cell number and cell differentials. Cytospin preparations were stained and evaluated microscopically for macrophages, polynuclear macrophages, polymorph nuclear neutrophils, lymphocytes, monocytes, eosinophils, and atypical cells. At least 400 cells were counted on each slide. The cell-free supernatant was collected to assess cell damage by measurement of total protein content and the release of lactate dehydrogenase (LDH) by Beckman Coulter autoanalyser Synchron LX20 (Beckman Coulter, Inc.) and to assess the induction of pro-inflammatory cytokines. The remainder of the supernatant was stored at −80°C.

### Measurement of pro-inflammatory cytokines

The presence of pro-inflammatory cytokines IL-1beta, IL-6, TNF-alfa, IFN-gamma, IL-13, GM-CSF, MCP-1, IL-12p70, IL-18, MIP-1a, MIP-2 and RANTES in the supernatant of the BALF was measured by a Bio-Plex Pro assay for rat cytokines according to manufacturer’s instructions (Bio-Rad Laboratories, Inc.).

### Measurement of oxidative stress

The induction of oxidative stress was determined by measuring the amount of reduced, oxidized and total glutathione in the lungs. The rinsed right lung was homogenized on ice in 4 ml of Phosphate/EDTA buffer (pH 7.5). Subsequently, the homogenate was centrifuged at 600 g for 10 minutes. To remove the proteins from the samples, the supernatant was mixed 1:1 with metaphosphorid acid, incubated for 15 minutes on ice, and centrifuged at 4000 rpm for 10 minutes. The amount of reduced, oxidized and total glutathione was measured by Beckmann Coulter Autoanalyser in the supernatant.

### Statistical analysis

The raw data of the BALF was corrected for retrieved fluid. To compare the different exposure groups, the data was analysed by the Kruskal Wallis nonparametric test (Graphpad Prism). Statistical significance is indicated with a * (*p =* < 0.05). In all graphs, error bars represent the standard deviation of the mean.

## Additional file

Additional file 1: Figure S1. SMPS and OPS particle size distribution (left) and CPC particle number concentration (right) of 15 nm and 410 nm silver particles.
