# Supplementary material for: Particle size dependent deposition and pulmonary inflammation after short-term inhalation of silver nanoparticles
Source: Part Fibre Toxicol. 2014 Sep 17;11:49. doi: 10.1186/s12989-014-0049-1 (PMC4410796; doi:10.1186/s12989-014-0049-1)
Supplement: Additional file 1: Figure S1. — SMPS and OPS particle size distribution (left) and CPC particle number concentration (right) of 15 nm and 410 nm silver particles. [file 12989_2014_49_MOESM1_ESM.docx]

**Additional file 1**

| 15 nm Ag | 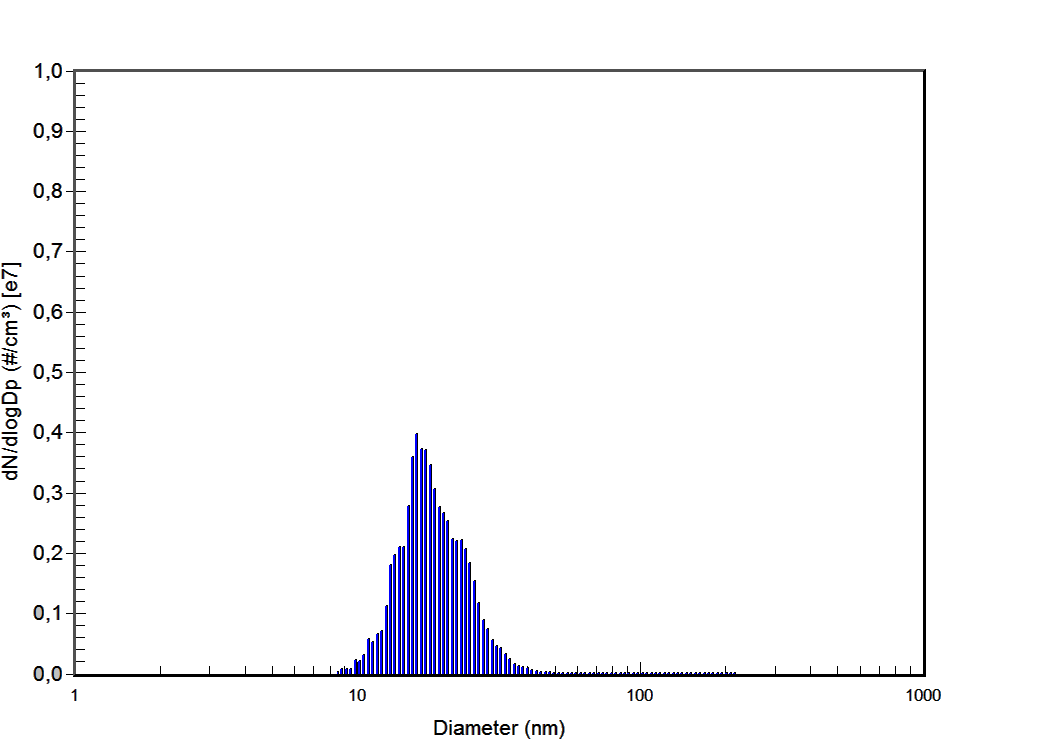 |  |
| --- | --- | --- |
| 410 nm Ag | 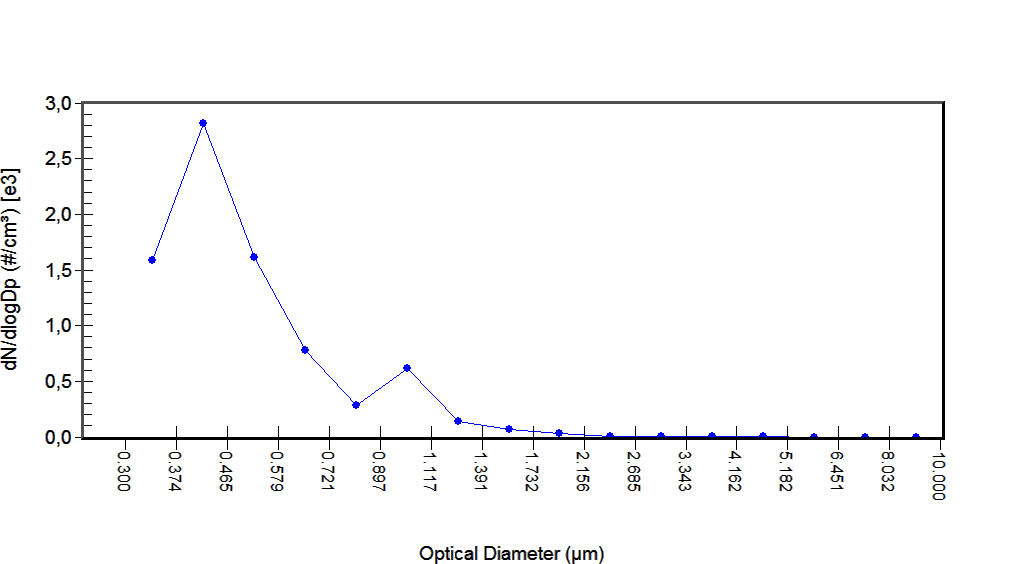 |  |

Figure S1. SMPS and OPS particle size distribution (left) and CPC particle number concentration (right) of 15 nm and 410 nm silver particles.
